# The Relationship Between Non-Invasive Tests and Digital Pathology for Quantifying Liver Fibrosis in MASLD

**DOI:** 10.3390/diagnostics15192475

**Published:** 2025-09-27

**Authors:** Xiaodie Wei, Lixia Qiu, Xinxin Wang, Chen Shao, Jing Zhao, Qiang Yang, Jun Chen, Meng Yin, Richard L. Ehman, Jing Zhang

**Affiliations:** 1 The Third Unit, Department of Hepatology, Beijing Youan Hospital, Capital Medical University, Beijing 100069, China; 2 Department of Pathology, Beijing Youan Hospital, Capital Medical University, Beijing 100069, China; 3 Department of Radiology, Beijing Youan Hospital, Capital Medical University, Beijing 100069, China; 4 Hangzhou Choutu Technology Co., Ltd., Hangzhou 310051, China; 5 Department of Radiology, Nanjing Drum Tower Hospital, Affiliated Hospital of Medical School, Nanjing University, Nanjing 210009, China; 6 Department of Radiology, Mayo Clinic, Rochester, MN 55905, USA

**Keywords:** metabolic dysfunction-associated steatotic liver disease, quantitative fibrosis parameters, liver fibrosis, magnetic resonance elastography, digital pathology

## Abstract

**Background:** It is crucial to evaluate liver fibrosis in metabolic dysfunction-associated steatotic liver disease (MASLD). Digital pathology, an automated method for quantitative fibrosis measurement, provides valuable support to pathologists by providing refined continuous metrics and addressing inter-observer variability. Although non-invasive tests (NITs) have been validated as consistent with manual pathology, the relationship between digital pathology and NITs remains unexplored. **Methods:** This study included 99 biopsy-proven MASLD patients. Quantitative-fibrosis (Q-Fibrosis) used second-harmonic generation/two-photon excitation fluorescence microscopy (SHG/TPEF) to quantify fibrosis parameters (q-FPs). Correlations between eight NITs and q-FPs were analyzed. **Results:** Using manual pathology as standard, Q-Fibrosis exhibited excellent diagnostic performance in fibrosis stages assessment with area under the receiver operating characteristic curves (AUCs) ranging from 0.924 to 0.967. In addition, magnetic resonance elastography (MRE) achieved the highest diagnostic accuracy (AUC: 0.781–0.977) among the eight NITs. Furthermore, MRE-assessed liver stiffness measurement (MRE-LSM) showed the strongest correlation with q-FPs, particularly adjusted by string length, string width, and the number of short and thick strings within the portal region. **Conclusions:** Both MRE and digital pathology demonstrated excellent diagnostic accuracy. MRE-LSM was primarily determined by collagen extent, location and pattern, which provide a new perspective for understanding the relationship between the change in MRE and histological fibrosis reverse.

## 1. Introduction

Metabolic dysfunction-associated steatotic liver disease (MASLD) has emerged as one of the leading causes of liver-related morbidity and mortality, with a global prevalence of 38% [1,2]. The pathological spectrum of MASLD ranges from metabolic dysfunction-associated steatotic liver (MASL) to metabolic dysfunction-associated steatohepatitis (MASH) with varying degrees of liver fibrosis, which may ultimately progress to cirrhosis [3,4]. Among these pathological features, liver fibrosis staging is the most critical determinant in predicting adverse outcomes in MASLD [5,6]. Consequently, an accurate assessment of fibrosis severity is crucial for determining prognosis.

Although manual histopathological assessment of liver biopsy is the gold standard for diagnosing liver fibrosis, it has several limitations, such as sampling error, intra-observer and inter-observer variability, and semi-quantitative staging system, etc. [7,8]. These drawbacks highlight the need for more precise, objective, and reproducible methods to evaluate liver fibrosis. Digital pathology has the potential to address these unmet needs and is currently an area of active investigation [9,10,11]. In recent years, second-harmonic generation and two-photon excitation fluorescence microscopy (SHG/TPEF) has emerged as a promising tool for quantifying liver fibrosis [12,13]. SHG/TPEF provides qFibrosis through a comprehensive and quantitative assessment of fibrosis, measuring the physical properties of collagen fibers, such as number, length, width, and direction [14,15]. Compared with conventional histology, SHG/TPEF improves the objectivity and reproducibility of fibrosis assessment. However, SHG/TPEF reliance on biopsy specimens limits its ability to overcome the inherent invasiveness and sampling variability of liver biopsy, particularly in fibrosis-related tissue heterogeneity. These limitations have driven the development of non-invasive tests (NITs), which aim to capture a larger volume of liver tissue and reduce sampling effects. Compared with ultrasound-based techniques, MR elastography assesses a larger proportion of the liver, which may potentially reduce sampling variability for longitudinal monitoring [16].

Non-invasive tests (NITs), such as vibration-controlled transient elastography (FibroScan^®^; Echosens, Paris, France), MRE, and serum biomarkers, demonstrated high consistency with manual pathology [17,18]. However, the relationship between NITs and q-FPs has not been explored. This study aimed to evaluate the correlation between q-FPs and NITs, attempting to provide a multidimensional assessment for diagnosing liver fibrosis.

## 2. Methods

### 2.1. Study Population

This was a retrospective study, which included consecutive 126 adult patients diagnosed with MASLD who underwent liver biopsy at Beijing Youan Hospital between 2022 and 2024. The diagnosis of MASLD was based on the latest Delphi criteria [19]. MASLD was diagnosed by liver biopsy as the presence of hepatic steatosis with at least one metabolic risk abnormalities after excluding other causes of steatosis. Metabolic abnormalities were defined as follows: (1) BMI ≥ 25 kg/m^2^ or waist circumference ≥ 90 cm in men and ≥80 cm in women; (2) blood pressure ≥ 130/85 mmHg or specific drug treatment; (3) Plasma triglycerides ≥ 1.70 mmol/L or specific drug treatment; (4) Plasma HDL-cholesterol ≤ 1.0 mmol/L for men and ≤1.3 mmol/L for women or specific drug treatment; (5) Prediabetes (fasting glucose levels 5.6 to 6.9 mmol/L or 2 h post-load glucose levels 7.8 to 11.0 mmol or HbA1c 5.7% to 6.4%). The Ethics Review Committee of Beijing Youan Hospital approved the study protocol (IRB number [2023]-052), and informed consent was obtained from all participants following Institutional Review Board guidelines.

### 2.2. MRI Measurements

Magnetic resonance imaging-proton density fat fraction (MRI-PDFF) and Magnetic resonance elastography (MRE) measurements were conducted by experienced radiologists using a GE 3.0 T scanner at our hospital’s radiology center. Patients fasted for a minimum of 4 h before the exam. Image processing of the entire liver and assessment of liver fat fraction was performed using GE ADW 4.7 workstation software. Liver stiffness was evaluated using MRE by placing an acoustic passive driver over the right anterior body wall and generating 60 Hz shear waves in the liver. The middle four sections of the liver were outlined, and measurements were averaged with a region of interest (ROI) >15 cm^2^ for each section. The radiologists were blinded to the patient’s clinical and pathological data.

### 2.3. Vibration-Controlled Transient Elastography (VCTE) Measurements

Patients were required to fast for at least 4 h before the examination. Controlled attenuation parameters (VCTE-CAP, dB/m) and liver stiffness measurement (VCTE-LSM, kPa) were assessed using Fibroscan (Echosens, Paris, France) with M or XL probes as appropriate. The test was considered reliable if the success rate exceeded 60% for a minimum of 10 valid measurements, and the interquartile range (IQR) to median ratio was below 30% [20]. Ultrasound physicians were blinded to the patient’s clinical and pathological data.

### 2.4. Clinical and Laboratory Data

Detailed patient information, including age, sex, height, weight, routine blood tests, biochemical tests, and medical history, was collected within 24 h prior to liver biopsy. The calculation formulas for non-invasive scores were as follows: MAST (Magnetic Resonance Imaging—AST score) = −12.17 + 7.07 log MRE + 0.037 PDFF + 3.55 log AST; FAST (FibroScan-AST score) = −1.65 + 1.07 × In (LSM) + 2.66 × 10^−8^ × CAP^3^—63.3 × AST^−1^; FIB-4 (Fibrosis-4 index) = Age × AST /platelets (10^9^/L) × ALT ^−1^; APRI (AST-to-Platelet Ratio Index) = (AST(U/L)/AST-ULN) × 100/platelets (10^9^ /L); AAR (AST-to-ALT Ratio) = AST(U/L)/ALT(U/L); NFS (NAFLD Fibrosis Score) = −1.675 + 0.037 × Age + 0.094 × BMI (kg/m^2^) + 1.13 × Glucose (mmol/L) + 0.99 × AST/ALT − 0.013 × platelets (10^9^/L) − 0.66 × Albumin (g/dL) [21,22,23,24,25].

### 2.5. Sample Preparation

The samples were prepared according to the following standard operating procedures. All liver tissue specimens were cut into 4–5 μm thick unstained sections for second-harmonic imaging (SHG). At the same time, some specimens were also cut for hematoxylin eosin (H&E) staining and Masson trichrome staining for histological evaluation.

### 2.6. Liver Biopsy

Liver biopsies were performed using a 16-gauge biopsy needle. The biopsy samples comprised one core of gray tissue measuring 2 mm in diameter and 20 mm in length, containing more than 10 portal tracts. The biopsy was usually performed on the anterior-superior or posterior-superior segment of the right lobe of the liver. Two experienced liver pathologists who did not obtain the clinical information conducted a histological evaluation using the NASH Clinical Research Network (NASH CRN) histological scoring system [3]. Mild and moderate and severe inflammation were defined as lobular inflammation 1 and 2–3. Fibrosis was graded as follows: F0 (no fibrosis), F1 (mild fibrosis, perisinusoidal or portal fibrosis), F2 (marked perisinusoidal and periportal fibrosis), F3 (advanced bridging fibrosis), and F4 (cirrhosis).

### 2.7. Two-Photon Microscopy/Second-Harmonic Generation

Unstained slices were scanned using the Genesis^®^ (HistoIndex, Singapore) automated multiphoton fluorescence imaging microscope to obtain high-quality digital images. Image quality was verified to ensure the absence of background noise, optimal brightness, and intact structural visualization. AI-based image analysis algorithms (HistoIndex, Singapore) were employed. Specimens were excited with a 780 nm laser, and signal acquisition included second-harmonic generation (SHG) at 390 nm and two-photon excitation fluorescence (TPEF) at 550 nm. Images were acquired at 20× magnification with a resolution of 512 × 512 pixels per 200 × 200 μm block.

The qFibrosis value is a continuous variable to accurately reflect differences in fibrosis severity. Regarding the calculation of the qF value: First, sequential feature selection was performed on the collagen parameters of training samples and their corresponding fibrosis stages to screen out key parameters; then, a linear regression model was trained using these selected parameters to finally generate the qF value. Before calculation, collagen parameters were first quantified based on tissue area and then normalized by dividing by their maximum value. In addition, the qF value was a dimensionless index and had undergone standardized processing to eliminate the influence of tissue size, ensuring comparability between samples.

### 2.8. Image Analysis

**Figure 1:**
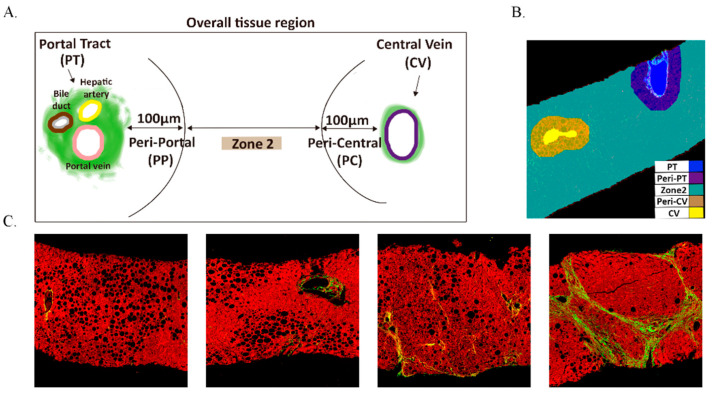
The distribution of overall tissue region and unstained liver biopsy samples are imaged by SHG/TPE fluorescence. (**A**) The definition of five regions; (**B**) the quantified image with five regions; Portal, Peri-Portal, Zone 2, Central Vein, and Peri-Central are denoted in blue, purple, green, brown, and yellow, respectively. (**C**) the SHG/TPEF imaging of different liver fibrosis stages. Collagen fiber is denoted in green.

AI-based identification of luminal structures in liver tissue uses SHG/TPEF imaging combined with machine learning for full-process automation, from signal acquisition to structure classification. SHG Channel Processing was as follows: First, Otsu’s thresholding method identifies collagen regions by distinguishing collagen from non-collagen tissues. Second, a “collagen segmentation” algorithm eliminates noise (collagen regions smaller than 15 μm^2^). Third, connected component analysis fills gaps and removes isolated collagen blocks, preserving large structures linked to blood vessels. Additionally, TPEF channel processing was as follows: First, Otsu’s thresholding identifies liver tissue regions (areas > 0.1 mm^2^) while excluding noise. Second, “Tissue segmentation” detects luminal structures (e.g., blood vessels, bile ducts, sinusoids, and fissures), labeled as “lumens” for exclusion from quantification. The Classification and Regression Tree (CART) algorithm combines SHG and TPEF results, identifying key anatomical landmarks (portal tract and central vein) to define functional zones (Zones 1, 2, and 3) in liver tissue.(Figure 1) The detailed descriptions of the parameters were provided in Supplementary Table S1.

### 2.9. Statistical Analysis

Continuous variables were expressed as means with standard deviations (SD) for normally distributed data and as medians with interquartile ranges (IQR) for non-normally distributed data. Categorical variables were presented as frequencies and percentages. Differences in liver fibrosis stages for qFibrosis were assessed using the Mann–Whitney U test. Post hoc pairwise comparisons between fibrosis stages were conducted with Bonferroni correction to evaluate in-between group differences. The correlation between q-FPs and liver fibrosis stages, as well as the NITs, was assessed using the Spearman rank correlation analysis. The diagnostic performance of q-FPs and NITs for liver fibrosis staging was evaluated using receiver operating characteristic (ROC) curve analysis, with the area under the receiver operating characteristic curve (AUC) used as a measure of diagnostic accuracy. The sensitivity, specificity, positive predictive value (PPV), and negative predictive value (NPV) were calculated for each diagnostic method at optimal cutoff values determined by the Youden index, 90% sensitivity, and 90% specificity, respectively. The agreement between q-FPs and pathologists’ liver fibrosis staging was assessed using the weighted Kappa. To identify the most important qFibrosis parameters associated with MRE and liver fibrosis staging, we used a random forest model to evaluate the relative importance of each q-FPs parameter. A two-tailed *p* ≤ 0.05 was considered statistically significant. All statistical analyses were conducted using the R package (version 4.2.1, http://cran.r-project.org/, accessed on 20 February 2024).

## 3. Results

### 3.1. Basic Characteristics of the Study Population

A total of 126 patients with suspected MASLD who underwent both liver biopsy and MRI between 2022 and 2024 were initially screened. Of these patients, 27 were excluded: 15 due to insufficient clinical data, 10 due to a biopsy–MRI interval longer than 6 months, and 2 due to concomitant liver diseases. Consequently, 99 patients with MASLD were finally included in the study (Supplementary Figure S1). As illustrated in Table 1, the mean age of the cohort was 39 years (IQR: 32–51), with 59.5% being male. Diabetes and hypertension were present in 28.3% of patients. The mean VCTE-CAP was 327 dB/m (SD: 40), and the median VCTE-LSM was 9.6 kPa (IQR: 7.3–11.8 kPa). Additionally, the median PDFF was 14.5% (IQR: 8.8–20.6%), while the median MRE-LSM was 2.99 kPa (IQR: 2.51–3.94 kPa). The mean value of q-FPs was 2.12 (SD: 0.95). The median values of MAST, FAST, FIB-4, APRI, NFS, and AAR were 0.13, 0.63, 1.02, 0.61, −2.45 and 0.66, respectively. In addition, post hoc analyses revealed significant in-between group differences for several variables. Specifically, MRE-LSM, VCTE-LSM, MAST, FAST, and qF values were significantly higher in patients with F3-4 (All *p* < 0.05). MRE-LSM, NFS, and qF values were significantly higher in patients with F4 compared with F0-3 (All *p* < 0.05). Liver fibrosis stages were distributed as follows: 17 patients (17.2%) were classified as F0, 16 patients (16.2%) as F1, 32 patients (32.3%) as F2, 28 patients (28.3%) as F3, and 6 patients (6.0%) as F4. MRE-LSM, VCTE-LSM, MAST, FAST, FIB-4, APRI, NFS, AAR, and qF values demonstrated significant differences with the increasing of liver fibrosis stages (all *p* < 0.001). AST and GGT increased significantly with increasing fibrosis stages. (*p* = 0.006 and 0.005, respectively) Additionally, there were statistically significant differences observed in the distribution of lobular inflammation and ballooning degeneration across different liver fibrosis groups (*p* < 0.001).

### 3.2. Accuracy of q-FPs and NITs in the Diagnosis of Liver Fibrosis

**Figure 2:**
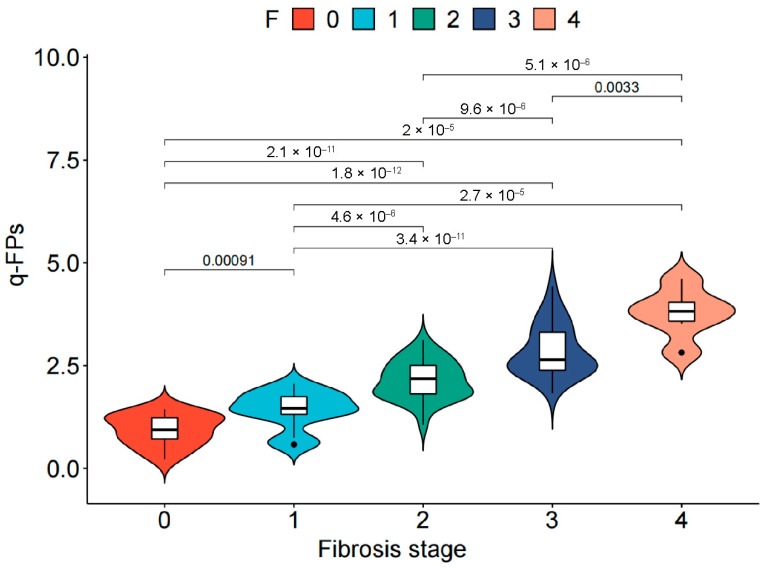
Distribution of qFibrosis in MASLD patients with different liver fibrosis stage. The white box in the middle represents data points in the 25–75% interquartile range, and the black line through the middle of the central white box represents the median value. Points represent outliers. MASLD, Metabolic dysfunction-associated steatotic liver disease.

**Figure 3:**
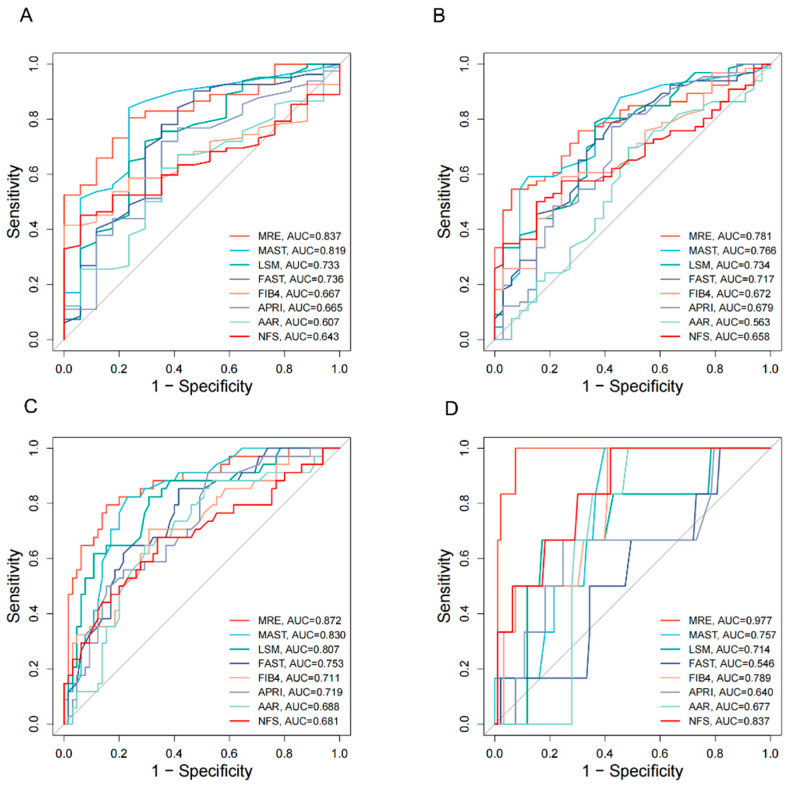
AUC plots of eight NITs for the diagnosis of liver fibrosis stage. (**A**) F1–4 vs. F0; (**B**) F2–4 vs. F0–F1; (**C**) F3–4 vs. F0–F2; (**D**) F4 vs. F0–F3. AUC, the area under the receiver operating characteristic curve; MRE, magnetic resonance elastography; MAST, magnetic resonance imaging-AST score; LSM, liver stiffness measurement; FAST, FibroScan-AST score; FIB4, Fibrosis-4 index; APRI, aspartate aminotransferase-to-platelet ratio; AAR, AST-to-ALT Ratio; NFS, NAFLD Fibrosis Score.

The q-FPs significantly increased with the severity of liver fibrosis (Figure 2). The AUCs (95%CI) of the q-FPs score for diagnosing each liver fibrosis stage were 0.961 (0.925, 0.996), 0.967 (0.937–0.997), 0.924 (0.875–0.973), and 0.955 (0.903–1.000), with cutoff values of 1.46, 1.74, 2.21, and 2.80, respectively. Among the eight NITs, MRE had the highest diagnostic efficacy for liver fibrosis, with AUCs of 0.837 (0.750–0.925), 0.781 (0.691–0.871), 0.872 (0.796–0.949), and 0.977 (0.946–1.000), respectively (Figure 3). Additionally, the cutoff values of MRE-LSM using Youden’s index were 2.57 kPa, 3.20 kPa, 3.24 kPa, and 4.31 kpa, respectively. The cutoff values of MRE-LSM calculated with 90% sensitivity were 2.23 kPa, 2.29 kPa, 2.64 kPa, and 4.35 kpa, respectively. The cutoff values of MRE-LSM calculated with 90% specificity were 2.88 kPa, 3.17 kPa, 3.62 kPa, and 4.23 kPa, respectively. Sensitivity, specificity, NPV, and PPV were presented in Table 2. Supplementary Table S2 showed that MRE-LSM cutoff values in the mild inflammation group were 2.17 kPa, 2.79 kPa, and 2.97 kPa, respectively. In the moderate-to-severe inflammation group, MRE-LSM cutoff values were 3.19 kPa, 3.87 kPa, and 4.31 kPa, respectively.

### 3.3. Correlation Between q-FPs Parameters and Liver Fibrosis Stages

A total of 186 q-FPs were measured, 36 of which were statistically significant. Among these, as shown in Table 3, PT, PeriPT, and ChickenWire parameters demonstrated significant positive correlations with the severity of liver fibrosis (the range of correlation coefficients ρ = 0.428–0.836, all *p* < 0.001). In contrast, parameters measured in Zone 2 exhibited a negative correlation with liver fibrosis (the range of correlation coefficients ρ = −0.502–−0.591, all *p* < 0.001). Among all the parameters, StrWidth and StrLength in the PT area exhibited the strongest correlations with liver fibrosis stages, with high correlation coefficients of 0.828 and 0.817, respectively. Notably, six PeriPT parameters showed correlation coefficients exceeding 0.80. Supplementary Table S3 showed that the inter group differences between liver histology and the distribution characteristics of important qFibrosis parameters are statistically significant (all *p* < 0.001).

### 3.4. Correlation Between q-FPs Parameters and Non-Invasive Testings

**Figure 4:**
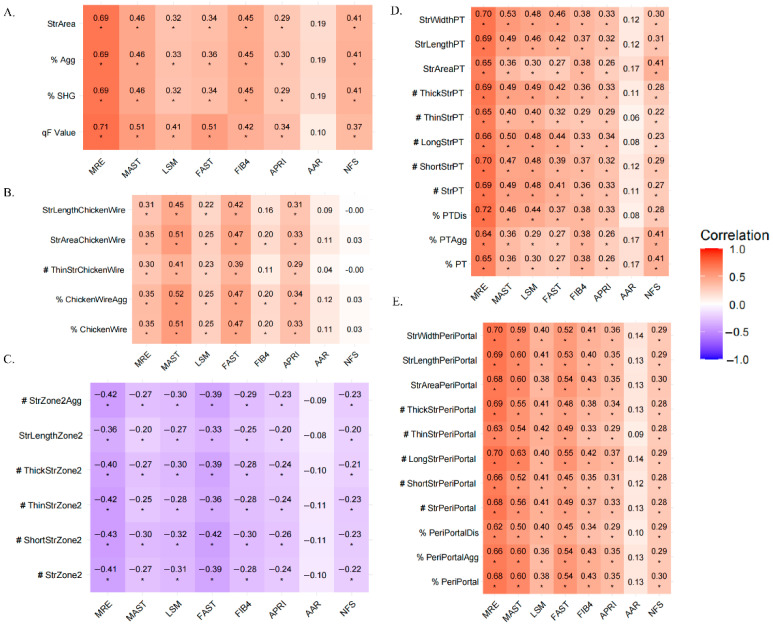
Heatmap of correlation coefficients between NITs and qFPs. The heatmap displays Spearman correlation coefficients between NITs and qFPs. The color gradient from orange to purple represents the correlation values, with purple indicating a negative correlation and orange indicating a positive correlation (* *p* < 0.05). # represents the number of the fiber parameter, % represent the proportion of the fiber parameter. A-E Show the relationship between NITs and qFPs in different regions: overall (**A**), Chickenwire (**B**), Zone 2 (**C**), Portal (**D**), and Periportal (**E**). PT, portal; MRE, Magnetic resonance elastography; LSM, Liver stiffness measurement; APRI, Aspartate aminotransferase-to-platelet ratio; AAR, Aspartate aminotransferase-to-alanine aminotransferase ratio; NFS, NAFLD Fibrosis Score.

**Figure 5:**
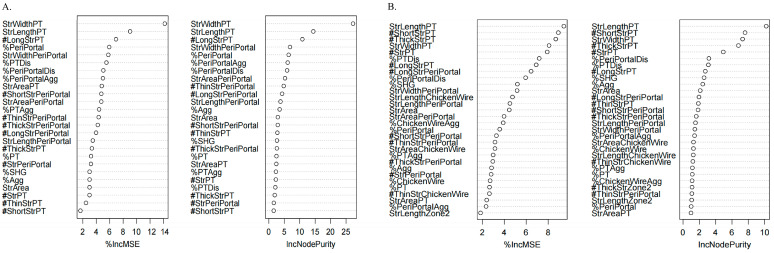
Random forest screening for parameters significantly related to fibrosis stages (**A**) and MRE (**B**). The plot showed the relative importance of features used in the random forest model. Importance was assessed using the decrease in mean square error (MSE) and Node Purity. MSE represented the importance of variables, and Node Purity represented the contribution of a feature to reducing prediction error within the random forest model. Higher MSE and Node Purity indicated greater feature importance.

We assessed the correlation between eight non-invasive liver fibrosis assessment methods and qFibrosis parameters. Figure 4 showed that MRE demonstrated the strongest correlation with parameters from the Overall, PT, and PeriPT. Using a random forest model to analyze feature importance, as demonstrated in Figure 5, six qFibrosis parameters most closely associated with MRE were identified: StrLengthPT, # ShortStrPT, #ThickStrPT, StrWidthPT, # StrPT, and %PTDis. Additionally, the most relevant parameters for liver fibrosis stages were StrWidthPT, StrLengthPT, #LongStrPT, %PeriPortal, StrWidthPeriPortal, and %PTDis.

## 4. Discussion

While digital pathology has demonstrated diagnostic advantages over manual methods, the consistency between NITs and digital pathology remained uncertain. Our study first identified significant correlations between MRE-LSM and q-FPs. MRE-LSM correlated with the fibrosis collagen string width, string length, and the number of thick and short strings in the portal region. This study reinforced the microstructural basis of MRE-assessed liver stiffness and further validated its strong performance.

Among all the NITs, MRE demonstrated the highest diagnostic efficacy for liver fibrosis. Previous meta-analysis reported AUC values of MRE for diagnosing F2, F3, and F4 are 0.92, 0.92 and 0.94, respectively, with cutoff values of 3.14, 3.53, and 4.45 kPa. Elevated levels of liver inflammation and GGT may affect the accuracy of MRE in diagnosing liver fibrosis [26]. However, in this study, the optimal cutoff values for MRE and other NITs, including VCTE, FIB-4, APRI, and NFS, were consistently lower than those recommended in the EASL-EASD-EASO guideline [27]. For example, guidelines typically propose cutoffs of 8 kPa for significant fibrosis and 12 kPa for advanced fibrosis. Similarly, widely accepted rule-out and rule-in cutoffs are 1.3 and 2.67 for FIB-4, 0.5 and 1.5 for APRI, and −1.455 and 0.676 for NFS in identifying advanced fibrosis. By contrast, our MASLD cohort had lower optimal cutoffs across these modalities. This discrepancy can be attributed to several factors. First, inflammation and elevated liver enzymes (AST and GGT), which varied significantly with fibrosis stage in our study, can increase stiffness or alter biochemical indices independently of fibrosis severity, thereby lowering diagnostic thresholds. Second, our patients were relatively younger and less obese than Western population, which may have influenced liver stiffness and biomarker performance. The population-specific characteristics, including ethnicity, may contribute to systematically lower optimal thresholds observed in Chinese patients. Therefore, these findings suggest that differences in inflammatory activity, metabolic profiles, and population characteristics across fibrosis stages may underlie the variability in diagnostic performance and the lower thresholds identified in our study.

With the growing demand for pathological diagnostic accuracy in new drug development, digital pathology has demonstrated unique advantages. HistoIndex developed a tissue imaging system which could acquire tissue signals from unstained slides. In order to ensure high image quality and reduce variability between different slices, this study adopted a strategy of device standardization and parameter fixation in the digital image acquisition process, ensuring that all devices and parameters were consistent, thereby maintaining consistency in signal sources and magnification between different slices and reducing differences in collagen fiber clarity caused by changes in devices or parameters [28,29,30]. The system could quantitatively analyze hundreds of individual collagen fiber parameters which were collectively referred to as q-FPs. The excellent diagnosis performance has been proved by previous studies [12,30,31,32,33,34], and validated again in our study. One of the international queues selected 17 parameters from 128 to construct the qFibrosis model. The AUC values for diagnosing F0–4 were 0.870, 0.881, 0.945, and 0.951, with cutoff values of 0.761, 0.882, 1.491, and 2.395, respectively [31]. As different studies varied depending on the selected features and patient cohorts, it is understandable that the threshold of qFPs in our study differs from other studies. Additionally, some specific q-FPs, such as string width (StrWidth) and string length (StrLength) in the portal region, exhibited the strongest correlations with liver fibrosis stages (correlation coefficient > 0.80). The findings were consistent with a study conducted by Wang et al. [12]. They found that the StrWidth and StrLength had a high accuracy in assessing liver fibrosis, with AUCs of 0.950 and 0.948. Interestingly, our analysis revealed differential correlation patterns between NITs and qFibrosis. In the periportal and pericentral regions, NITs strongly correlated with qFibrosis. However, a negative correlation was observed specifically in zone 2. This can be explained by the progression of liver fibrosis, where collagen morphology evolves toward cirrhosis-like features, forming bridging connections and even fusions of portal tracts and central veins [34]. These results highlight qFibrosis can capture subtle pathological features across different liver zones.

A key limitation in fibrosis diagnosis lay in the discordance between whole-liver MRE and biopsy assessments. The discordance observed between whole-liver MRE and biopsy assessments can be primarily attributed to sampling volume inconsistent. A single 16-gauge core represents only a localized tract, whereas MRE reflects the mechanical properties of the entire liver. This disparity is further amplified by spatial heterogeneity of fibrosis. At the macroscopic level, paired biopsies from different sites have been reported to exhibit histologic discordance of ≥1 stage in approximately 40% of cases [35]. At the microscopic level, regional differences in fibrosis regression and tissue stiffness have been linked to zonated stellate-cell activity and distinct collagen architectures [36]. As a result, biopsy-based validation remains inherently vulnerable to spatial heterogeneity. While digital pathology enhances reproducibility and provides quantitative metrics, it cannot eliminate the intrinsic sampling bias imposed by small-volume cores. Therefore, we explicitly acknowledge that sampling bias between focal biopsy and whole-liver method is a fundamental limitation of all biopsy-anchored validation and should temper interpretation of discordance.

This study has some limitations. First, it was conducted in a single-center cohort with a relatively small sample size, which necessitates further validation in larger, multi-center cohorts. Second, the study was focused on patients with MASLD, and the result might be different in other chronic liver diseases.

In conclusion, the results of this study demonstrated relationships between MRE-based liver stiffness measurements and liver structural parameters revealed by digital pathology. These relationships may provide a basis for better understanding the biomechanical effects of specific histological patterns of liver fibrosis. Further studies are needed to explore correlations between digital pathology and NITs, as well as to understand the limitations imposed on histopathology by sampling effects, in order to further define the role of NITs as alternatives to liver biopsy.

## Figures and Tables

**Table 1:** Baseline Characteristics of Study Population according to the fibrosis stages.

Variable	Total (*n* = 99)	F0 (*n* = 17)	F1 (*n* = 16)	F2 (*n* = 32)	F3 (*n* = 28)	F4 (*n* = 6)	*p* Value
Clinical characteristics							
Male, *n* (%)	59 (59.60)	14 (82.35)	10 (62.50)	17 (53.12)	14 (50.00)	4 (66.67)	0.243
Age, years	39 (32, 51)	38 (32, 42)	37 (29, 44)	46 (33, 55)	38 (32, 59)	56 (40, 63)	0.136
BMI, kg/m^2^	27.9(26.1, 30.2)	28.8 (25.4, 30.1)	27.1 (25.8, 29.6)	27.7 (26.3, 29.3)	28.1 (26.0, 33.8)	28.5(27.4,29.8)	0.835
HT, *n* (%)	28 (28.28)	4 (23.53)	2 (12.50)	7 (21.88)	11 (39.29)	4 (66.67)	0.075
Diabetes, *n* (%)	28 (28.28)	3 (17.65)	4 (25.00)	10 (31.25)	7 (25.00)	4 (66.67)	0.266
Laboratory tests							
ALT, U/L	81 (57, 123)	67 (53, 98)	73 (44, 116)	89 (65, 126)	99 (71, 125)	59 (51, 67)	0.238
AST, U/L	53 (40, 88)	39 (33, 46)	58 (37, 72)	50 (42, 89)	68 (53, 107) ^a^*	51 (41, 58)	0.006
TBIL, µmol/L	17.42 (6.35)	19.20 (6.01)	18.29 (7.88)	16.17 (5.80)	17.02 (6.69)	18.62 (3.63)	0.532
ALB, g/L	47 (44, 49)	48 (46, 49)	48 (44, 50)	47 (45, 49)	45 (44, 48)	45 (41, 49)	0.485
GGT, U/L	56 (34, 91)	38 (31, 56)	49 (34, 78)	40 (31, 69)	72 (55, 97)	96 (70, 135)	0.030
HB, g/L	149 (13)	155 (9)	150 (12)	148 (14)	148 (12)	143 (18)	0.259
PLT, 10^9^/L	241 (67)	247 (52)	281 (69)	244 (53)	226 (76)	167 (68) ^a$^	0.005
Glucose, mmol/L	5.5 (5.0, 6.4)	5.2 (4.9, 5.8)	5.0 (4.5, 5.6)	6.0 (5.3, 6.5) ^b#^	5.5 (5.0, 6.8)	6.6 (6.0, 6.7) ^b$^	0.007
HbA1c, %	5.9 (5.6, 6.6)	5.7 (5.5, 6.1)	5.8 (5.3, 5.9)	6.2 (5.8, 6.9)	6.0 (5.7, 6.8)	6.7 (6.2, 6.9)	0.021
TC, mmol/L	5.10 (1.01)	5.32 (0.70)	5.41 (1.17)	4.96 (0.96)	5.14 (1.07)	4.28 (1.03)	0.143
TG, mmol/L	1.63 (1.13, 2.38)	1.76 (1.20, 1.92)	1.55 (1.07, 2.27)	1.73 (1.16, 2.77)	1.60 (1.15, 2.38)	1.08(0.92, 1.39)	0.410
HDL-C, mmol/L	1.02 (0.86, 1.21)	0.98 (0.86, 1.20)	0.94 (0.90, 1.01)	1.07 (0.91, 1.23)	1.03 (0.77, 1.20)	1.26 (1.15, 1.40)	0.049
LDL-C, mmol/L	3.36 (1.02)	3.41 (0.69)	3.76 (0.93)	3.12 (1.05)	3.56 (1.11)	2.52 (0.92)	0.051
Imaging examinations							
PDFF, %	14.5 (8.8, 20.6)	12.4 (6.0, 19.3)	14.1 (10.6, 16.8)	16.6(10.4, 21.4)	15.0 (8.9, 20.7)	6.9 (6.4, 11.9)	0.219
MRE-LSM, kPa	2.99 (2.51, 3.94)	2.44 (2.17, 2.55)	2.77 (2.52, 3.15)	2.89 (2.47, 3.26)	3.98 (3.23, 4.27) ^abc^*	5.13 (4.88, 5.31) ^abc$^	<0.001
CAP, dB/m	327 (40)	320 (44)	328 (38)	327 (46)	332 (31)	324 (40)	0.904
VCTE-LSM, kPa	9.6 (7.3, 11.9)	7.4 (5.7, 8.4)	7.7(6.3, 10.1)	8.5(7.5, 10.1)	12.0(9.9,17.8)^abc^*	14.6(10.6,16.5) ^a$^	<0.001
Non-invasive tests							
MAST	0.13 (0.05, 0.29)	0.03 (0.02, 0.05)	0.10 (0.05, 0.16)	0.10 (0.06, 0.22)	0.29 (0.18,0.46) ^abc^*	0.27 (0.19, 0.38) ^a$^	<0.001
FAST	0.63 (0.43, 0.77)	0.39 (0.30, 0.64)	0.55 (0.42, 0.66)	0.60 (0.50, 0.72)	0.77 (0.63, 0.86) ^abc^*	0.67 (0.50, 0.72)	<0.001
FIB-4	1.02 (0.63, 1.88)	0.77 (0.62, 0.94)	0.92 (0.43, 2.05)	0.94 (0.61, 1.51)	1.43 (0.80, 2.86)	2.27 (1.24, 3.62) ^a$^	0.005
APRI	0.61 (0.42, 1.10)	0.43 (0.35, 0.69)	0.53 (0.33, 0.76)	0.58 (0.42, 0.88)	0.95 (0.56, 1.29) ^a^*	1.13 (0.58, 1.39)	0.006
NFS	−2.46 (1.66)	−3.13 (0.74)	−3.08 (1.49)	−2.60 (1.61)	−1.94 (1.82)	−0.45 (1.62) ^abc$^	0.001
AAR	0.66 (0.53, 0.90)	0.57 (0.52, 0.78)	0.70 (0.51, 1.07)	0.60 (0.52, 0.73)	0.82 (0.61, 1.10)	0.86 (0.80, 0.87)	0.028
qF Value	2.12 (0.95)	0.94 (0.38)	1.42 (0.45)	2.14 (0.47) ^ab#^	2.86 (0.63) ^abc^*	3.78 (0.60) ^abc$^	<0.001
Liver histology							
Steatosis grade, *n* (%)							0.496
1	26 (26.26)	8 (47.06)	5 (31.25)	7 (21.88)	4 (14.29)	2 (33.33)	
2	38 (38.38)	5 (29.41)	6 (37.50)	12 (37.50)	12 (42.86)	3 (50.00)	
3	35 (35.35)	4 (23.53)	5 (31.25)	13 (40.62)	12 (42.86)	1 (16.67)	
Lobular inflammation, *n* (%)							<0.001
1	47 (47.47)	17 (100.00)	12 (75.00)	12 (37.50)	4 (14.29)	2 (33.33)	
2	43 (43.43)	0 (0.00)	4 (25.00)	17 (53.12)	18 (64.29)	4 (66.67)	
3	9 (9.09)	0 (0.00)	0 (0.00)	3 (9.38)	6 (21.43)	0 (0.00)	
Hepatocyte ballooning, *n* (%)							<0.001
0	20 (20.20)	12 (70.59)	3 (18.75)	4 (12.50)	0 (0.00)	1 (16.67)	
1	59 (59.60)	5 (29.41)	11 (68.75)	20 (62.50)	21 (75.00)	2 (33.33)	
2	20 (20.20)	0 (0.00)	2 (12.50)	8 (25.00)	7 (25.00)	3 (50.00)	

#, *, and $ represent the *p* < 0.05 after Bonferroni correction for multiple comparisons with F2, F3, and F4, respectively. a, b, and c represent the comparisons of F0 vs. F2–4, F1 vs. F2–4, F2 vs. F3–4, respectively. Continuous variables were expressed as mean ± SD or median (IQR) as appropriate. BMI, Body Mass Index; PDFF, Proton Density Fat Fraction; MRE, Magnetic Resonance Elastography; VCTE, Vibration Controlled Transient Elastography; CAP, Controlled Attenuation Parameter; LSM, Liver Stiffness Measurement; MAST, Magnetic resonance imaging-AST Score; FAST, FibroScan-AST score, FIB-4, Fibrosis-4 index; APRI, AST-to-Platelet Ratio Index; NFS, NAFLD Fibrosis Score; AAR, AST-to-ALT Ratio; qF, Quantitative Fibrosis; TC, Total Cholesterol; TG, Triglycerides; HDL-C, High-Density Lipoprotein Cholesterol; LDL-C, Low-Density Lipoprotein Cholesterol; ALT, Alanine Aminotransferase; AST, Aspartate Aminotransferase; TBIL, Total Bilirubin; ALB, Albumin; GGT, Gamma-Glutamyl Transferase; HB, Hemoglobin; PLT, Platelet Count; HbA1c, Hemoglobin A1c.

**Table 2:** Diagnostic performance of qFibrosis and NITs for liver fibrosis staging.

Fibrosis Stage		qFibrosis	MRE	MAST	VCTE	FAST	FIB4	APRI	AAR	NFS
F0 VS. F1–4	AUC (95%CI)	0.961 (0.925–0.996)	0.837 (0.750–0.925)	0.819 (0.702–0.936)	0.733 (0.594–0.871)	0.736 (0.592–0.880)	0.667 (0.558–0.777)	0.665 (0.519–0.812)	0.607 (0.465–0.748)	0.643 (0.532–0.755)
	Cutoff (Youden)	1.46	2.57	0.06	7.85	0.40	1.40	0.52	0.62	−2.155
	Se (%)	89	81	84	72	90	41	72	62	45
	Sp (%)	100	77	77	71	53	100	65	65	94
	NPV (%)	65	45	50	34	53	26	32	26	26
	PPV (%)	100	94	94	92	90	100	91	90	97
	Cutoff (90%Se)	1.42	2.23	0.04	6.05	0.38	0.48	0.34	0.44	−4.54
	Sp (%)	88	29	59	35	47	12	18	6	0
	NPV (%)	65	94	97	95	96	85	90	72	0
	PPV (%)	97	21	31	22	26	17	19	16	16
	Cutoff (90%Sp)	1.30	2.88	0.16	10.5	0.69	1.35	0.90	0.93	−2.31
	Se (%)	95	66	54	39	40	45	38	26	46
	NPV (%)	79	93	90	87	88	89	87	85	89
	PPV (%)	98	54	49	41	41	44	40	31	45
F0–1 VS. F2–4	AUC (95%CI)	0.967 (0.937–0.997)	0.781 (0.691–0.871)	0.766 (0.665–0.868)	0.734 (0.629–0.839)	0.717 (0.607–0.826)	0.672 (0.562–0.781)	0.679 (0.560–0.798)	0.563 (0.438–0.689)	0.658 (0.552–0.764)
	Cutoff	1.74	3.20	0.17	7.85	0.54	1.08	0.52	0.59	−2.155
	Se (%)	94	55	59	79	79	59	77	70	50
	Sp (%)	88	94	88	64	58	76	58	52	85
	NPV (%)	88	51	52	60	58	48	56	46	46
	PPV (%)	94	95	91	81	79	83	79	74	87
	Cutoff (90%Se)	1.80	2.29	0.04	6.35	0.41	0.54	0.40	0.44	−4.26
	Sp (%)	91	21	48	33	36	21	36	9	15
	NPV (%)	64	91	96	95	94	91	94	81	87
	PPV (%)	98	19	26	22	23	19	23	17	18
	Cutoff (90%Sp)	1.78	3.17	0.17	11.05	0.80	2.12	1.42	1.24	−1.62
	Se (%)	91	55	55	38	29	26	14	11	36
	NPV (%)	67	91	91	88	86	86	84	83	87
	PPV (%)	98	55	55	46	40	37	24	19	45
F0–2 VS. F3–4	AUC (95%CI)	0.924 (0.875–0.973)	0.872 (0.796–0.949)	0.830 (0.749–0.911)	0.807 (0.714–0.899)	0.753 (0.656–0.851)	0.711 (0.601–0.820)	0.719 (0.615–0.822)	0.688 (0.578–0.798)	0.681 (0.563–0.799)
	Cutoff	2.21	3.24	0.17	9.65	0.59	1.18	0.52	0.76	−2.585
	Se (%)	97	79	82	82	85	71	91	65	68
	Sp (%)	78	85	77	69	58	69	48	71	66
	NPV (%)	98	89	89	88	88	82	91	79	80
	PPV (%)	70	73	65	58	52	55	48	54	51
	Cutoff (90%Se)	2.22	2.64	0.08	6.90	0.42	0.60	0.42	0.44	−4.260
	Sp (%)	78	43	48	29	31	23	37	11	14
	NPV (%)	65	96	96	94	94	90	95	85	88
	PPV (%)	95	25	27	21	21	19	23	17	18
	Cutoff (90%Sp)	2.52	3.62	0.32	11.05	0.82	2.21	1.24	1.21	−0.760
	Se (%)	68	68	41	62	32	35	29	12	35
	NPV (%)	36	93	88	92	87	87	86	83	87
	PPV (%)	97	57	44	54	42	40	36	18	40
F0–3 VS. F4	AUC (95%CI)	0.955 (0.903–1.000)	0.977 (0.946–1.000)	0.757 (0.623–0.892)	0.714 (0.492–0.937)	0.546 (0.306–0.785)	0.789 (0.632–0.947)	0.640 (0.373–0.907)	0.677 (0.569–0.784)	0.837 (0.695–0.979)
	Cutoff	2.8	4.31	0.17	12.75	0.4	1.18	1.04	0.66	−2.585
	Se (%)	100	100	100	67	100	100	67	100	100
	Sp (%)	85	93	60	83	18	59	75	52	58
	NPV (%)	100	100	100	98	100	100	97	100	100
	PPV (%)	30	46	14	20	7	14	15	12	13
	Cutoff (90%Se)	2.82	4.35	0.17	6.90	0.41	1.19	0.42	0.67	−2.540
	Sp (%)	85	92	63	23	19	59	22	54	58
	NPV (%)	51	96	95	87	85	95	86	94	94
	PPV (%)	96	70	32	18	18	30	18	27	29
	Cutoff (90%Sp)	3.17	4.23	0.51	18.50	0.86	2.88	1.58	1.29	−0.160
	Se (%)	83	100	17	0	17	50	17	0	50
	NPV (%)	53	100	84	81	84	90	84	81	90
	PPV (%)	98	68	26	0	31	52	26	0	52

MRE, Magnetic Resonance Elastography; VCTE, Vibration Controlled Transient Elastography; MAST, Magnetic resonance imaging-AST Score; FAST, FibroScan-AST score; FIB4, Fibrosis-4 index; APRI, AST-to-Platelet Ratio Index; NFS, NAFLD Fibrosis Score; AAR, AST-to-ALT Ratio; AUC, Area Under the Curve; Se, Sensitivity; Sp, Specificity; NPV, Negative Predictive Value; PPV, Positive Predictive Value.

**Table 3:** The correlation between qFibrosis parameters and Fibrosis stages.

Parameter	Correlation Coefficient (rhρ)	*p* Value
qFibrosis Value	0.836	<0.001
StrWidthPT	0.828	<0.001
StrLengthPT	0.817	<0.001
%PeriPortal	0.814	<0.001
StrAreaPeriPortal	0.814	<0.001
StrWidthPeriPortal	0.814	<0.001
StrLengthPeriPortal	0.811	<0.001
%PeriPortalAgg	0.808	<0.001
#StrPeriPortal	0.804	<0.001
#ThickStrPeriPortal	0.800	<0.001
#LongStrPeriPortal	0.799	<0.001
#ShortStrPeriPortal	0.785	<0.001
#LongStrPT	0.784	<0.001
#ThinStrPeriPortal	0.784	<0.001
%PeriPortalDis	0.784	<0.001
#ThickStrPT	0.761	<0.001
#StrPT	0.761	<0.001
#ShortStrPT	0.729	<0.001
%PTDis	0.721	<0.001
#ThinStrPT	0.681	<0.001
%Agg	0.677	<0.001
%SHG	0.653	<0.001
StrArea	0.653	<0.001
%PT	0.642	<0.001
StrAreaPT	0.642	<0.001
%PTAgg	0.639	<0.001
%ChickenWireAgg	0.502	<0.001
%ChickenWire	0.493	<0.001
StrAreaChickenWire	0.493	<0.001
StrLengthChickenWire	0.442	<0.001
#ThinStrChickenWire	0.428	<0.001
StrLengthZone2	−0.502	<0.001
#ThinStrZone2	−0.536	<0.001
#ThickStrZone2	−0.564	<0.001
#StrZone2	−0.566	<0.001
#StrZone2Agg	−0.588	<0.001
#ShortStrZone2	−0.591	<0.001

PT, portal tract; Rhρ was calculated according to Spearman’s method. # represents the number of the fiber parameter, % represent the proportion of the fiber parameter.

## Data Availability

The datasets used during the current study are available from the corresponding author on reasonable request.

## Supplementary Materials

The following supporting information can be downloaded at: https://www.mdpi.com/article/10.3390/diagnostics15192475/s1, Figure S1: Flow diagram of study enrollment in patients with suspected MASLD underwent liver biopsy; Table S1: The descriptions of qFibrosis parameters; Table S2: Diagnostic efficacy of MRE-LSM for diagnosing liver fibrosis in different subgroups of inflammation; Table S3: Distribution characteristics of important qFibrosis parameters related to MRE and liver histology in different liver fibrosis stages.

## Abbreviations

MASLD, metabolic dysfunction-associated steatotic liver disease; MASL, metabolic dysfunction-associated steatotic liver; MASH, metabolic dysfunction-associated steatohepatitis; NITs, non-invasive tests; MAST, magnetic resonance imaging-AST score; FAST, FibroScan-AST score; FIB-4, Fibrosis-4 index; APRI, AST-to-Platelet Ratio Index; AAR, AST-to-ALT Ratio; NFS, NAFLD Fibrosis Score; SHG/TPEF, second-harmonic generation/two-photon excitation fluorescence microscopy; MRE-LSM; magnetic resonance elastography–liver stiffness measurement; AUC, area under the receiver operating characteristic curve; VCTE, vibration-controlled transient elastography; ROI, region of interest; BMI, Body Mass Index; PDFF, Proton Density Fat Fraction; HDL-C, High-Density Lipoprotein Cholesterol; LDL-C, Low-Density Lipoprotein Cholesterol; ALT, Alanine Aminotransferase; AST, Aspartate Aminotransferase; GGT, Gamma-Glutamyl Transferase; PT, portal tract; PeriPT, periportal tract.
