# Supplementary material for: The Relationship Between Non-Invasive Tests and Digital Pathology for Quantifying Liver Fibrosis in MASLD
Source: Diagnostics (Basel). 2025 Sep 27;15(19):2475. doi: 10.3390/diagnostics15192475 (PMC12524164; doi:10.3390/diagnostics15192475)
Supplement: Supplementary file 1 [file diagnostics-15-02475-s001.zip › diagnostics-3854491-supplementary.pdf]

Supplymentary materials

Supplymentary Table 1. The descriptions of qFibrosis parameters.

Regions	Parameters	Descriptions
	%Agg	Percentage of aggregated collagen for overall fibrosis in tissue area
Overall	%SHG	Percentage of total collagen for overall fibrosis in tissue area
	StrArea	Area of all strings for overall fibrosis unit tissue area
	StrWidthPT	Width of all strings for portal tract fibrosis per unit tissue area
	StrLengthPT	Length of all strings for portal tract fibrosis per unit tissue area
	#LongStrPT	Number of long strings for portal tract fibrosis per unit tissue area
	#ThickStrPT	Number of thick strings for portal tract fibrosis per unit tissue area
	#StrPT	Number of strings for portal tract fibrosis per unit tissue area
Portal	#ShortStrPT	Number of short strings for portal tract fibrosis per unit tissue area
	%PTDis	Percentage of distributed collagen for portal tract fibrosis in tissue area
	#ThinStrPT	Number of thin strings for portal tract fibrosis per unit tissue area
	%PT	Percentage of total collagen for portal tract fibrosis in tissue area
	StrAreaPT	Area of all strings for portal tract fibrosis per unit tissue area
	%PTAgg	Percentage of aggregated collagen for portal tract fibrosis in tissue area
	%PeriPortal	Percentage of total collagen for peri-portal fibrosis in tissue area
	StrAreaPeriPortal	Area of all strings for peri-portal fibrosis per unit tissue area
	StrWidthPeriPortal	Width of all strings for peri-portal fibrosis per unit tissue area
Periportal	StrLengthPeriPortal	Length of all strings for peri-portal fibrosis per unit tissue area
	%PeriPortalAgg	Percentage of aggregated collagen for peri-portal fibrosis in tissue area
	#StrPeriPortal	Number of strings for peri-portal fibrosis per unit tissue area
	#ThickStrPeriPortal	Number of thick strings for peri-portal fibrosis per unit tissue area

	#LongStrPeriPortal	Number of long strings for peri-portal fibrosis per unit tissue area
	#ShortStrPeriPortal	Number of short strings for peri-portal fibrosis per unit tissue area
	#ThinStrPeriPortal	Number of thin strings for peri-portal fibrosis per unit tissue area
	%PeriPortalDis	Percentage of distributed collagen for peri-portal fibrosis in tissue area
ChickenWire	%ChickenWireAgg	Percentage of aggregated collagen for chickenwire fibrosis in tissue area
	%ChickenWire	Percentage of total collagen for chickenwire fibrosis in tissue area
	StrAreaChickenWire	Area of all strings for chickenwire fibrosis per unit tissue area
	StrLengthChickenWire	Length of all strings for chickenwire fibrosis per unit tissue area
	#ThinStrChickenWire	Number of thin strings for chickenwire fibrosis per unit tissue area
Zone2	StrLengthZone2	Length of all strings for zone 2 fibrosis per unit tissue area
	#ThinStrZone2	Number of thin strings for zone 2 fibrosis per unit tissue area
	#ThickStrZone2	Number of thick strings for zone 2 fibrosis per unit tissue area
	#StrZone2	Number of strings for zone 2 fibrosis per unit tissue area
	#StrZone2Agg	Number of aggregated strings for zone 2 fibrosis per unit tissue area
	#ShortStrZone2	Number of short strings for zone 2 fibrosis per unit tissue area

Supplymentary Table 2. Diagnostic efficacy of MRE-LSM for diagnosing liver fibrosis in different subgroups of inflammation.

Fibrosis stages	Subgroups	Cutoff	Sensitivity	Specificity	NPV	PPV
F0 VS F1-4	Mild inflammation	2.17	57	82	52	85
	Moderate and severe inflammation	-	-	-	-	-
F0-1 VS F2-4	Mild inflammation	2.79	61	76	76	61
	Moderate and severe inflammation	3.19	64	75	15	97
F0-2 VS F3-4	Mild inflammation	2.97	83	83	97	42
	Moderate and severe inflammation	3.87	68	88	70	86
F0-3 VS F4	Mild inflammation	-	-	-	-	-
	Moderate and severe inflammation	4.31	100	85	100	36

MRE, Magnetic Resonance Elastography； LSM, Liver Stiffness Measurement; NPV, Negative Predictive Value; PPV, Positive Predictive Value.

Supplymentary Table 3. Distribution characteristics of important qFibrosis parameters related to MRE and liver histology in different liver fibrosis stages.

Variables	Total (n = 99)	F0 (n = 17)	F1 (n = 16)	F2 (n = 32)	F3 (n = 28)	F4 (n = 6)	P value
StrLengthPT	1533.13 (1051.11, 2543.25)	681.41 (552.23,1036.34)	1069.19 (732.93,1318.34)	1550.42 (1288.19,2133.42)	2814.01 (2236.55,3435.92)	3819.33 (3506.44,4422.29)	< 0.001
StrWidthPT	636.71 (437.59, 1042.90)	338.79 (215.19,409.86)	448.63 (298.44,498.13)	667.66 (512.89,855.98)	1141.75 (859.79,1487.82)	1517.66 (1318.15,1692.70)	< 0.001
%PTDis	0.01 (0.01, 0.02)	0.01 (0.00,0.01)	0.01 (0.00,0.01)	0.01 (0.01,0.01)	0.02 (0.01,0.03)	0.03 (0.03,0.04)	< 0.001
#StrPT	12.05 (6.03, 18.30)	4.86 (3.12,7.37)	5.65 (4.73,7.40)	12.36 (6.79,16.06)	19.41 (15.47,30.73)	30.35 (26.96,40.37)	< 0.001
#ShortStrPT	6.80 (2.86, 10.97)	2.54 (2.08,3.96)	2.86 (2.03,4.18)	6.89 (3.12,9.24)	10.97 (8.94,18.66)	22.55 (18.85,26.79)	< 0.001
#LongStrPT	4.83 (2.84, 8.03)	2.18 (1.66,2.89)	2.84 (2.43,3.16)	5.04 (3.73,6.94)	8.86 (6.33,11.83)	9.09 (6.82,13.58)	< 0.001
#ThickStrPT	10.73 (5.35, 16.86)	4.42 (3.03,6.47)	4.99 (4.20,6.61)	10.93 (5.58,13.19)	17.13 (13.83,27.76)	26.92 (24.30,36.92)	< 0.001
StrLengthPT	1533.13 (1051.11, 2543.25)	681.41 (552.23,1036.34)	1069.19 (732.93,1318.34)	1550.42 (1288.19,2133.42)	2814.01 (2236.55,3435.92)	3819.33 (3506.44,4422.29)	< 0.001
StrWidthPT	636.71 (437.59, 1042.90)	338.79 (215.19,409.86)	448.63 (298.44,498.13)	667.66 (512.89,855.98)	1141.75 (859.79,1487.82)	1517.66 (1318.15,1692.70)	< 0.001
%PeriPortal	0.95 (0.56, 1.32)	0.42 (0.26,0.57)	0.52 (0.43,0.68)	0.93 (0.68,1.18)	1.46 (1.22,1.96)	1.46 (1.17,1.68)	< 0.001
StrWidthPeriPortal	2065.17	979.11	1288.95	2035.70	3128.17	3223.65	< 0.001

	(1385.56, 2951.80)	(691.70,1557.17)	(1093.05,1713.67)	(1662.42,2683.52)	92812.11,4178.48)	(2992.71,3730.96)

PT, Portal; MRE, Magnetic Resonance Elastography.

Supplymentary Figure 1. Flowchart of patient selection for the study. MASLD, Metabolic dysfunction-associated steatotic liver disease; MRI, Magnetic resonance imaging.

Figure S1. Flow diagram of study enrollment in patients with suspected MASLD underwent liver biopsy.

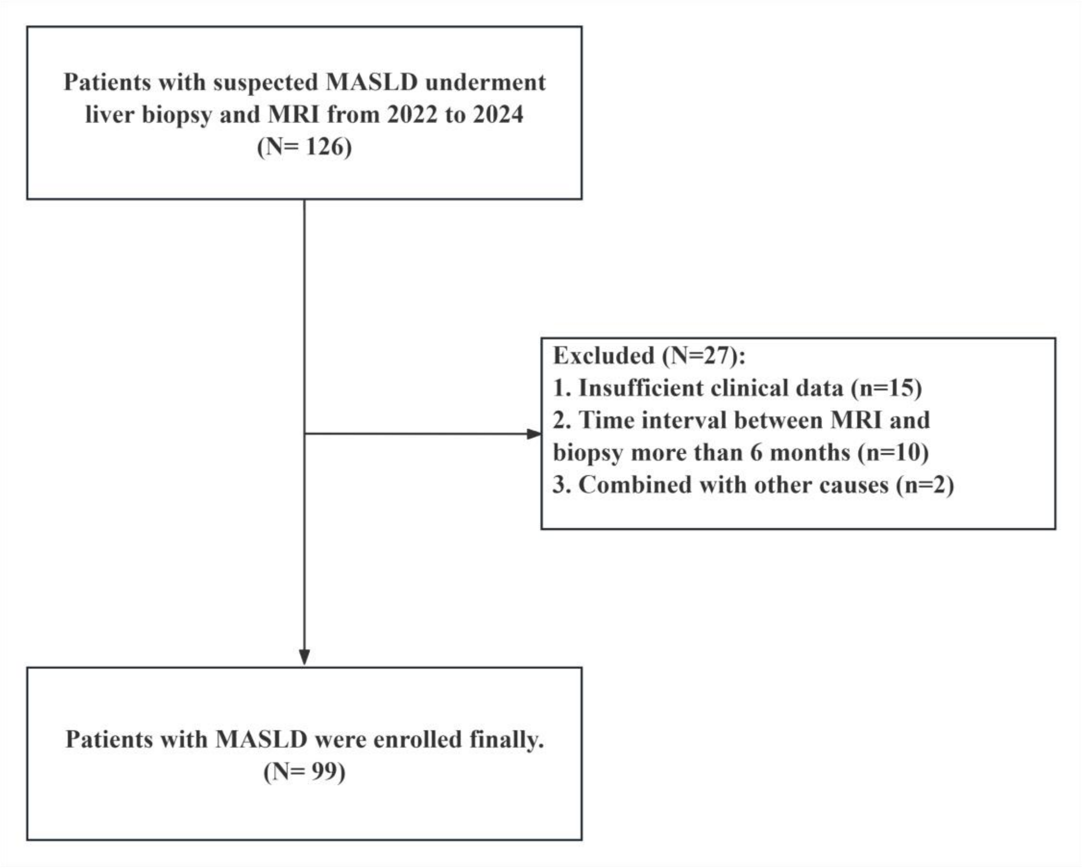

MASLD, metabolic dysfunction-associated steatotic liver disease.
